# Passive-elastic knee-ankle exoskeleton reduces the metabolic cost of walking

**DOI:** 10.1186/s12984-020-00719-w

**Published:** 2020-07-27

**Authors:** Ettore Etenzi, Riccardo Borzuola, Alena M. Grabowski

**Affiliations:** 1 Beckett Thermal Solutions S.r.l, Formigine (MO), Italy; 2 Department of Movement, Human and Health Sciences, University of Rome “Foro Italico”, Rome, Italy; 3 Department of Integrative Physiology, University of Colorado, Boulder, Boulder, CO USA; 4 Department of Veterans Affairs Eastern Colorado Healthcare System, Aurora, CO USA

**Keywords:** Biomechanics, Locomotion, Energetic cost, Wearable robotics

## Abstract

**Background:**

Previous studies have shown that passive-elastic exoskeletons with springs in parallel with the ankle can reduce the metabolic cost of walking. We developed and tested the use of an unpowered passive-elastic exoskeleton for walking that stores elastic energy in a spring from knee extension at the end of the leg swing phase, and then releases this energy to assist ankle plantarflexion at the end of the stance phase prior to toe-off. The exoskeleton uses a system of ratchets and pawls to store and return elastic energy through compression and release of metal springs that act in parallel with the knee and ankle, respectively. We hypothesized that, due to the assistance provided by the exoskeleton, net metabolic power would be reduced compared to walking without using an exoskeleton.

**Methods:**

We compared the net metabolic power required to walk when the exoskeleton only acts at the knee to resist extension at the end of the leg swing phase, to that required to walk when the stored elastic energy from knee extension is released to assist ankle plantarflexion at the end of the stance phase prior to toe-off. Eight (4 M, 4F) subjects walked at 1.25 m/s on a force-measuring treadmill with and without using the exoskeleton while we measured their metabolic rates, ground reaction forces, and center of pressure.

**Results:**

We found that when subjects used the exoskeleton with energy stored from knee extension and released for ankle plantarflexion, average net metabolic power was 11% lower than when subjects walked while wearing the exoskeleton with the springs disengaged (*p* = 0.007), but was 23% higher compared to walking without the exoskeleton (*p* < 0.0001).

**Conclusion:**

The use of a novel passive-elastic exoskeleton that stores and returns energy in parallel with the knee and ankle, respectively, has the potential to improve the metabolic cost of walking. Future studies are needed to optimize the design and elucidate the underlying biomechanical and physiological effects of using an exoskeleton that acts in parallel with the knee and ankle. Moreover, addressing and improving the exoskeletal design by reducing and closely aligning the mass of the exoskeleton could further improve the metabolic cost of walking.

## Introduction

Reducing the metabolic cost of walking through use of assistive devices such as exoskeletons would allow humans to walk further with less effort and fatigue and could allow those with physical disabilities to be able to walk. The idea of creating mechanical devices to assist human movement and reduce metabolic cost has been around since the year 1890 [1]. During the twentieth century, many scientists have focused their efforts on creating mechanical devices that reduce the metabolic cost of human movement [2], and during the last decade, use of an unpowered passive-elastic exoskeleton has reduced the metabolic cost of walking compared to walking without an exoskeleton by improving efficiency (quotient of mechanical and metabolic power) [3, 4].

Distinct biomechanical tasks needed to walk, such as supporting body weight and redirecting/accelerating the center of mass, require leg muscle force and work, and thus incur a metabolic cost. The single limb support phase of walking has been modelled as an inverted pendulum [5–7]. In this model, the body’s mass is represented by a point mass and the stance leg by a rigid massless strut [6, 7]. During the single support phase, mechanical energy is conserved through the phasic exchange of kinetic and gravitational energy. However, the muscles of the leg must produce force to support body weight during single support and thus require metabolic energy [8]. The muscles of the leg must also generate mechanical work to transition body mass from step to step during the double support phase and this incurs a greater metabolic cost than body weight support [8–10]. Redirecting the center of mass during the step-to-step transition requires approximately 45% of the overall net metabolic power; whereas supporting body weight requires approximately 28% of the overall net metabolic power needed for steady-speed level-ground walking [8]. To facilitate walking, the muscles surrounding the ankle, knee, and hip joints dissipate and generate mechanical work; these changes in negative and positive energy could be exploited by a passive-elastic exoskeleton to reduce metabolic cost.

**Fig. 1:**
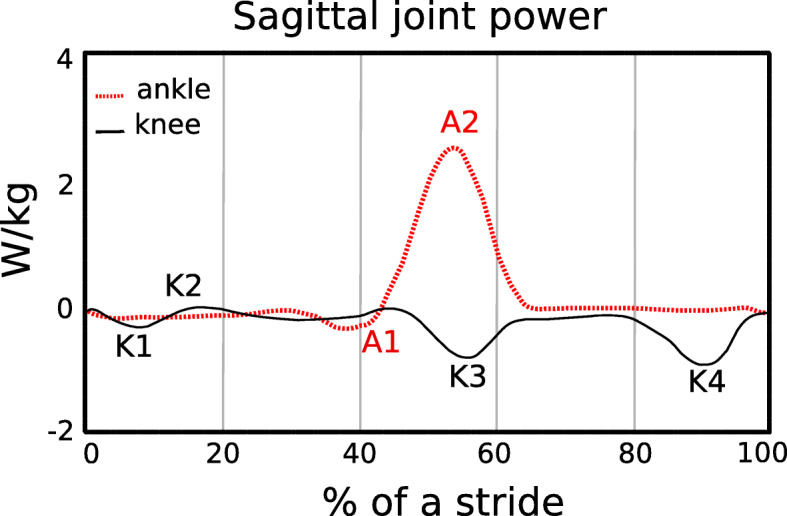
Average sagittal plane ankle (A), and knee (K) joint mechanical power during level ground walking at 1.10 m/s over a stride for one leg, starting at heel strike. Data are from a previous study [13]. Negative peak power regions for the ankle and knee joints are denoted as A1 and K1, K3, and K4, respectively. Mechanical power and thus energy, is dissipated/absorbed during negative ankle (A1) and knee (K4) joint minimums [11]. At ~ 35–40% of the stride, the ankle plantar-flexors contract eccentrically to control ankle joint dorsiflexion. During terminal swing (K4), the hamstrings contract eccentrically to slow the speed of the swinging leg and avoid knee hyperextension just prior to the subsequent heel-strike (~ 90% of the stride). Positive mechanical power regions are labelled as A2 and K2 and correspond to the concentric contraction of the ankle plantar-flexors during late stance and the knee extensors during early stance, respectively

The muscles surrounding the ankle joint are primarily responsible for absorbing/producing power to facilitate the redirection of the center of mass during the step-to-step transition [11]. Over a stride, the muscles surrounding the knee joint dissipate or absorb/store net negative mechanical power and work, whereas the muscles surrounding the ankle and hip joints generate net positive mechanical power and work [12]. Negative and positive peaks in joint power indicate when mechanical energy is absorbed and generated, respectively, during a stride (Fig. 1). Negative peak power indicates eccentric contraction of the ankle plantar-flexor muscles from heel-strike through tibial progression, knee extensor muscles during heel-strike, rectus femoris during late stance, and biceps femoris during late stance (Fig. 1). Positive power regions primarily correspond to concentric contraction of the ankle plantar-flexor muscles during late stance, knee extensor muscles during early stance and hip flexor muscles during early swing phase. All these muscle contractions incur a metabolic cost. Thus, an exoskeleton that stores energy corresponding with the eccentric contraction of the knee extensor muscles and returns this energy during the concentric contraction of the ankle plantar-flexor muscles could decrease the metabolic cost of walking.

In order to reduce the metabolic cost of walking, use of an exoskeleton should not alter kinematic gait parameters such as stride length and step width, or kinetic parameters such as ground reaction forces. Previous studies have shown that when people walk with stride lengths and stride frequencies different from preferred, the metabolic cost of walking increases [14–16]. Walking speed is the product of stride length and stride frequency. At a fixed walking speed, the relationship between stride frequency and metabolic cost is represented by a U–shaped curve with the minimum metabolic cost corresponding to the preferred stride frequency [17]. Similarly, previous studies show that when humans walk with wider or narrower step widths compared to preferred, their metabolic cost increases [18–20]. Step width indicates the lateral distance between the midlines of the feet [21]. At a fixed walking speed, metabolic cost increases with the square of step width [19]. Thus, use of an exoskeleton that results in changes to stride length, stride frequency and step width compared to preferred could increase the metabolic cost of walking.

The development of wearable devices such as exoskeletons has been motivated by the challenge to reduce the metabolic cost of walking. In 1890, Nicholas Yagn conceptualized and received a patent for the first exoskeleton for assisting walking, running, and jumping using pneumatically powered gas bags [1]. Since then, many investigators have developed electrically powered or battery-powered lower limb exoskeletons for medical applications, neurorehabilitation therapy, augmentation, and military use [2, 22–25]. Most of the recent powered exoskeletons use actuators to provide assistance at the ankle joint during powered plantarflexion at the end of the stance phase of walking [25–30]. Specifically, use of powered exoskeletons has reduced the muscle activity and lower limb joint work needed by the user during level-ground walking compared to wearing the exoskeleton with the power turned off. Together with the timing of the assistance, the weight of these devices (12 kg to 38 kg [22]) may be one of the reasons why use of a powered exoskeleton does not decrease metabolic cost compared to normal walking without any wearable system [24].

With new methodological innovations, current research shows that exoskeletons can improve the metabolic cost of walking. Malcolm et al. [30] used optimal actuation timing predicted by a mathematical model combined with a tethered electrically-powered exoskeleton that assists ankle plantarflexion, and found that use of the exoskeleton reduced the metabolic cost of walking at 1.38 m/s by 6.0 ± 2.0% (mean ± SD) compared to walking without the exoskeleton. Mooney et al. [25] developed a battery-powered exoskeleton that utilizes a mathematical model to control the magnitude of positive mechanical power provided by the exoskeleton during ankle powered plantarflexion, and reduced metabolic cost by 8 ± 3% compared to walking without an exoskeleton at 1.5 m/s. Thus, through the optimal timing and magnitude of applied power, use of a powered exoskeleton can reduce the metabolic cost of walking.

The way that an exoskeleton is attached to a person can affect the assistance provided to the person and thus the metabolic cost of walking. Panizzolo et al. [31] have investigated the use of an exosuit equipped with compliant textiles that provide assistance instead of rigid structures, such as those used in other powered exoskeletons. This approach aimed to improve the interface between the exoskeleton and the body [32] and reduce the mass on distal body segments to have less of an effect on metabolic cost [33, 34]. In particular, the exosuit was designed to provide assistance during both ankle joint plantarflexion at the end of the stance phase and hip joint flexion during the early swing phase. Using this exosuit, net metabolic power in the powered condition was 14.2 ± 6.1% lower than in the unpowered condition, but net metabolic power was not reduced with respect to normal walking at 1.5 m/s.

Use of passive-elastic exoskeletons has reduced the metabolic cost of walking by enhancing the mechanism of elastic energy storage and return at the ankle joint, with springs in parallel to the Achilles tendon [3, 25]. Recent studies demonstrate that storing and returning elastic energy during the phases of ankle joint negative and positive mechanical power can significantly reduce metabolic cost [3, 35]. Collins et al. [3] has shown that use of a passive-elastic exoskeleton in parallel with the ankle joint that stores and returns energy during the negative and positive phases of ankle joint power (Fig. 1) reduced metabolic cost by 7.2 ± 2.6% (mean ± SD) compared to walking without an exoskeleton at 1.25 m/s. Many others have investigated how use of a passive-elastic exoskeleton, which does not require an external power supply and is not equipped with sensors or actuators, affects walking [3, 36–39]. Panizzolo et al. [3] demonstrated that it is possible to reduce the metabolic cost of walking by more than 3% with a passive device that assists the hip joint compared to normal walking. Rome et al. [38] found that use of rubber bands in parallel with the hip joint can reduce the metabolic costs of carrying loads during walking, whereas Dean et al. [39] has shown that the use of a two-joint passive-elastic exoskeleton that works in parallel with the hip and knee joints can reduce the activity of lower limb muscles compared to normal walking [39].

We aimed to determine if a passive-elastic exoskeleton in parallel with the knee and ankle joints could reduce the metabolic cost of walking. We built an exoskeleton that stores energy from knee extension during the late leg swing phase, which corresponds to negative peak knee power (Fig. 1, K4) since it represents the greatest magnitude of energy absorption/storage during the stride [12]. Then, we designed the exoskeleton to release the energy stored from knee extension to assist ankle powered plantarflexion, which corresponds to positive peak ankle power during late stance (Fig. 1, A2). We designed our experiments to test three hypotheses. First, we hypothesized that the use of a passive-elastic exoskeleton that resists knee extension during the late leg swing phase would reduce metabolic power during level-ground walking compared to walking without an exoskeleton. Second, we hypothesized that the use of a passive-elastic exoskeleton that stores energy from knee extension during the late leg swing phase and returns energy for ankle powered plantarflexion during late stance would reduce metabolic power during level-ground walking compared to walking without an exoskeleton. Third, we hypothesized that the use of a passive-elastic exoskeleton would not change stride length, ground contact time, peak ground reaction forces, and step width during level ground walking compared to walking without an exoskeleton.

## Material and methods

### Participants

Eight healthy subjects [4 M and 4 F, mean ± SD age: 25 ± 3 years, mass: 73 ± 15 kg, height: 174 ± 10 cm, standing leg length: 83 ± 5 cm] participated in the study. We measured their leg lengths from the greater trochanter to the medial malleolus and averaged the right and left leg lengths. All subjects gave informed written consent before participating according to the University of Colorado Boulder Institutional Review Board.

We measured metabolic rates, ground reaction forces, and center of pressure while subjects walked on a dual-belt force measuring treadmill with the exoskeleton springs disengaged (no springs), engaged in parallel with the knee only, engaged in parallel with the knee and ankle, engaged in parallel with the knee only but with a longer engagement rope length, and engaged in parallel with the knee and ankle but with a longer engagement rope length, and without the exoskeleton.

### Description of the exoskeleton

**Fig. 2:**
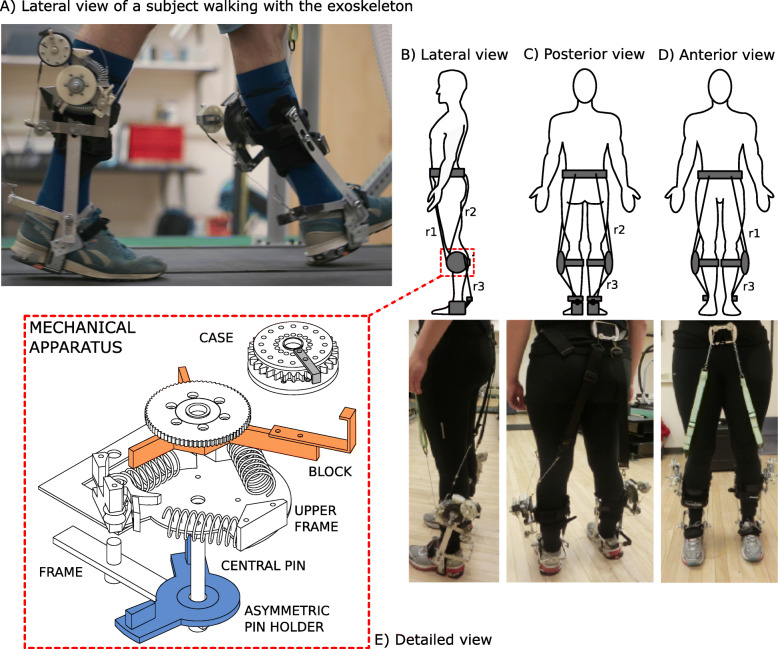
**a** Lateral view of a subject walking on the treadmill while using the exoskeleton just as the rope (r3) provides plantarflexion assistance to the trailing leg at the end of the stance phase. **b** Lateral, **c** Posterior, and **d** Anterior views of the exoskeleton attached to the body and corresponding pictures of a subject wearing the exoskeleton with ropes attached from the anterior belt to the apparatus (r1), posterior belt to the apparatus (r2) and apparatus to the ankle frame (r3). E) An exploded view of the mechanical apparatus. We designed the upper frame so that it could engage up to three linear springs. We used two springs during the experimental sessions in order to attain an angular spring stiffness of 17.45 Nm/rad. The mechanical apparatus is comprised of a frame that anchors the upper frame to the braces on the shank. That frame provides a central pin for the rotation of the asymmetric pin holder and block. The case rotates around a pin (not shown), fixed on the longer arm of the block

We custom-made the passive-elastic exoskeleton, which consists of a lightweight aluminum frame secured to the lower leg with a modified knee brace (Ottobock HealthCare LP, Austin, US). The exoskeleton is equipped with a mechanical apparatus comprised of six parts (Fig. 2, panel e). The primary frame has a central pin that is fixed on the external side of a modified knee brace, and all the other parts are attached to this frame. An asymmetric pin holder includes two small pins and rotates around the central pin, and an upper frame is fixed to the primary frame and holds the system of springs and a pawl. A block, with three arms and a ratchet wheel rotates about the central pin and compresses the system of springs on the upper frame. A case, which contains a spiral spring is attached distally on the longest arm of the block. In addition, the exoskeleton includes two inextensible ropes (r1, r2) that are fixed proximally on the anterior and posterior side of a belt worn by the subject positioned just above the iliac spines and glutei (Fig. 2), and distally to the exoskeletal mechanical apparatus on the pawl (r1) and the asymmetric pin holder (r2). Specifically, r2 is comprised of two parts (Fig. 2b). The superior part consists of a 4 cm wide piece of nylon webbing that lies posteriorly over the middle of the gluteus, whereas the lower part is a nylon rope that is attached to the mechanical apparatus. 4 cm wide pieces of nylon webbing were used to increase the surface area on the skin and prevent potential discomfort related to the pressure exerted by the rope on the skin. A third inextensible rope (r3) that originates from the exoskeletal case, is attached to the ankle frame surrounding the subject’s heel (Fig. 2a). The total mass of the exoskeleton is ~ 1.4 kg per leg.

**Fig. 3:**
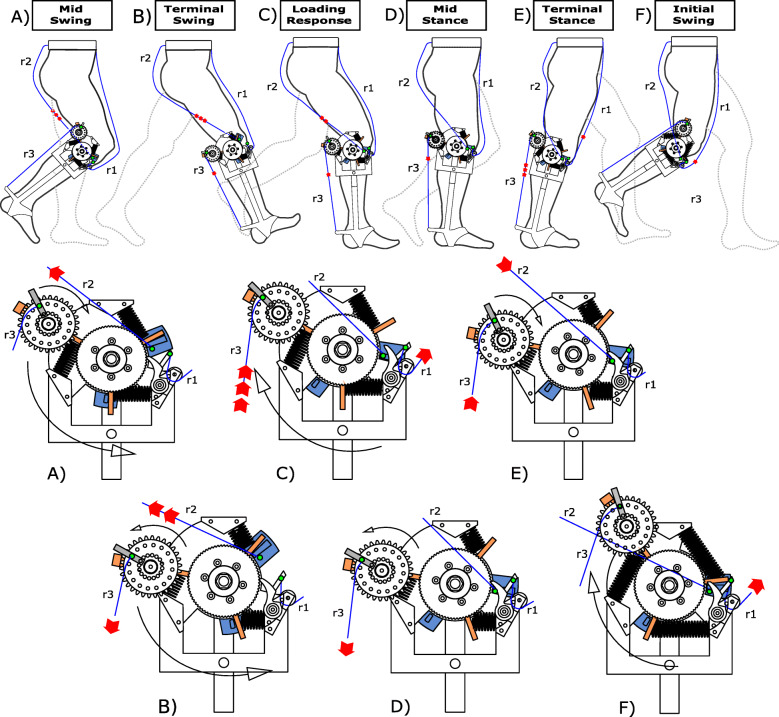
Engagement and disengagement of the exoskeleton mechanical apparatus during different phases of a walking stride. The upper portion of the figure shows the action of the exoskeleton during a stride and the lower portion provides a detailed view of the knee mechanism. Red arrows indicate the direction that ropes r1, r2 and r3 are moving during a specific phase (in panel E, red arrows are also used to indicate the extension of the linear springs). **a** During Mid-Swing, r2 begins to stretch due to knee extension and pulls the central pin. This knee extension movement continues until (**b**) Terminal Swing, and results in the rotation of the block which, in turn, compresses the springs. r3 also undergoes tension and causes the counterclockwise rotation of the case during Terminal Swing (considering the lateral side of the right leg). **c** During the Loading Response, both r2 and r3 become slack as the hip extends, which causes clockwise rotation of the central pin and case, respectively. **d** During Mid-Stance, r3 is stretched again as the hip rotates over the ankle and the case is locked so that there is no additional counterclockwise rotation. **e** During Terminal Stance, r1 is stretched due to hip extension, which pulls the pawl out of the ratchet, and allows the linear springs to extend and release their stored elastic energy. In this way, the block rotates clockwise and pulls on r3, which provides assistance during ankle powered plantarflexion. **f** At the subsequent Initial Swing only r1 is still stretched, while all of the other components of the device are disengaged

We 3D printed plastic ratchets and a pawl that were attached to the lateral portion of the brace and allowed energy from the movement of the knee to be stored through compression of the metal springs. The exoskeleton is attached to each leg, but we describe the effects of the exoskeleton for the right leg (Fig. 3). At the end of the swing phase, after hip extension is maximal, r2 is tensioned and rotates the asymmetric pin holder counter-clockwise (Fig. 3a), which pushes the block against the system of springs and compresses them (Fig. 3b). At the same time, the 3D-printed pawl engages the ratchet wheel to keep the spring compressed and prevent the clockwise rotation of the block (Fig. 3b, c, d). As r2 slackens, the asymmetric pin holder returns to its original position and the knee can freely flex from the loading response through mid-stance without any interaction with the engaged mechanical apparatus. Prior to the beginning of the experimental trial, we set the length of r2 to ensure it only tensioned in late swing, at maximum hip flexion. As the shank moves forward, due to its inertia, the tension in rope r2 provides a force that compresses the springs and allows the exoskeleton to decelerate the shank prior to heel strike. Also, the biological ankle’s motion is not affected by the exoskeleton due to a spiral spring inside the case. This spring allows the case to rotate from mid-swing through mid-stance (Fig. 3a, b, c, d), while r3 remains slack and does not interfere with the motion of the ankle. r3 only undergoes tension during late stance, when the hip extends, and the case rotation is locked. During this phase, just prior to powered plantarflexion, r1 is extended along the anterior side of the upper leg, disengages the pawl, and releases the compressed springs (Fig. 3e). The pawl disengagement allows the system of springs to release the stored energy; the block rotates clockwise lifting the case attached to its longer arm, which pulls up on r3 and assists the ankle joint during the push–off phase (Fig. 3f). The posterior case, attached to the longer arm of the block, rotates freely during a stride except for at the end of the stance phase, when the central ratchet is released. Ankle joint plantarflexion is therefore assisted by the elastic energy return of the exoskeleton. The exoskeleton does not constrain the biological range of motion of the ankle joint, and it is positioned at the same height and just lateral to the ankle, which connects the brace and heel frame of the exoskeleton. The ankle frame does not include a bearing but is designed to have low friction and the lever arm of the ankle frame is approximately 0.125 m.

### Spring stiffness

For each leg, we used two linear springs with a combined linear compression stiffness of 654.2 N/m within the exoskeleton. The number of springs and their stiffness were determined prior to the experimental trials based on a pilot study of 2 subjects, as described below. We compared different stiffness configurations to determine the configuration that resulted in the lowest metabolic power and consistent engagement of the spring-ratchet-pawl mechanism. The rotation of the block compresses the springs, and the stiffness and energy return of the exoskeleton results from the spring angular stiffness. We measured angular stiffness (k) of the springs using a three-dimensional motion capture system (Vicon 512 System, Oxford, UK) at 100 Hz with reflective markers placed on the ends of the springs that were compressed using masses of 0 to 10.9 kg. Total angular stiffness was 17.45 Nm/rad for the exoskeletal configuration with two springs. This stiffness resists knee extension at the end of the swing phase.

### Walking trials

Subjects were asked to walk on a dual-belt force-measuring treadmill (Bertec, Columbus, Ohio, USA) at 1.25 m/s. We used the same adjustable exoskeleton for all subjects. The dimensions of the exoskeleton were designed for subjects with standing leg lengths of 75.5–90.4 cm. For this reason, we measured each subject’s standing leg lengths prior to participation to ensure that the device would fit. The exoskeleton was secured to each subject’s foot and superior portion of his or her shank using five Velcro straps. r1 and r2 were then attached posteriorly and anteriorly to the belt worn by the subjects. Prior to experimental trials, anthropometric data were collected from the lower limb (leg, thigh, and shank lengths). These measurements were used to determine the length of r2 since it directly affected the engagement of the spring-ratchet-pawl mechanism. In addition, we used a high-speed camera (Casio EXILIM EX-ZR1000, Tokyo, Japan) at 480 Hz to record several initial strides of each subject with the device attached and enabled. Using the video, we adjusted the timing of energy release from the springs within a stride by adjusting the length of r1. The ideal timing for energy storage was estimated from the video, where the engagement of the springs started during terminal swing phase just prior to heel strike. We also visually verified that the springs released during ankle powered plantarflexion. Prior to experimental trials, subjects walked during three separate 7-min adaptation trials for a total of 21 min wearing the exoskeleton. Then, subjects performed a total of seven 7-min trials. First, subjects stood in place quietly while we measured their metabolic rates. Then, subjects completed six trials walking at 1.25 m/s without the exoskeleton and wearing the exoskeleton while we measured their metabolic rates, ground reaction forces, and center of pressure. Subjects walked using two different posterior rope lengths (r2-long and r2-short), where the short length was 1 cm shorter than the long length determined from the video, to analyze the effectiveness of a smaller/greater engagement of the spring-ratchet-pawl mechanism. For each rope length, two trials were performed, one with r3 attached (ankle and knee assistance enabled) and one without r3 attached (knee assistance only). Finally, subjects performed a walking trial wearing the exoskeleton, but with no assistance, such that the springs were disengaged. All trials were 7 min long in order to obtain steady-state metabolic rates, 7 trials were performed per session and were separated by at least 3 min rest to account for any potential effects of fatigue, and the trial order was randomized to account for any potential adaptation effects [6].

### Metabolic rate

Subjects were at least 2 h post-prandial prior to beginning experimental trials. We measured rates of oxygen consumption and carbon dioxide production throughout each trial using indirect calorimetry (Parvo Medics True One 2400, Sandy, Utah, USA). Then, we averaged the rates of oxygen consumption and carbon dioxide production during minutes 3–5 of each trial to ensure steady-state metabolic rates [8]. We then visually ensured that each subject reached steady-state metabolic rates and used the average rates to calculate metabolic power using a standard equation [40]. Net metabolic power was calculated by subtracting metabolic power during standing from gross metabolic power during walking. We ensured that the respiratory exchange ratio (RER) for each subject was lower than 1.1 during each trial, and therefore assumed that subjects primarily used oxidative metabolism. We normalized metabolic rates to each subject’s body mass.

### Ground reaction forces

We measured ground reaction forces (GRFs) using a dual-belt force-measuring treadmill (Bertec Corp., Columbus, OH) at 1000 Hz for thirty seconds during minutes 3 and 4 of each trial. A low pass 1st order Butterworth filter with a 30 Hz cut off was used to decouple the low frequency component of the horizontal and vertical GRFs. Then, we used a custom Matlab code (Matlab 2015b, Mathworks Inc., Natick, MA, United States) to calculate stride length, ground contact time, and peak horizontal and vertical GRFs from the filtered GRFs. We identified ground contact using a 40 N vertical GRF threshold and used this to calculate stride time. We calculated step width using the medio-lateral center of pressure from 25 to 75% of ground contact. We determined step width as the average distance between the feet using medio-lateral center of pressure from at least 20 steps.

### Statistics

Statistical analysis was performed using IBM SPSS 24.0 (IBM Corp., Armonk, NY, USA). We performed one-way repeated measures ANOVAs to detect significant differences in net metabolic power, stride length, ground contact time, peak horizontal and vertical ground reaction forces, and step width with Condition (six-levels) as a within-subject factor. When a significant main effect of Condition was identified by the overall ANOVAs, we made five orthogonal planned comparisons of: 1. the exoskeleton engaged versus exoskeleton disengaged, 2. the exoskeleton versus no exoskeleton, 3. the exoskeleton engaged with longer versus shorter posterior ropes, 4. the exoskeleton with ankle assistance engaged versus no ankle assistance, and 5. the exoskeleton with longer posterior ropes and ankle assistance (best exoskeletal condition) versus the exoskeleton disengaged. We used an SPSS Bonferroni-corrected *p*-value for multiple comparisons (SPSS, Armonk, NY). SPSS adjusts the initial *p-*value by multiplying it by the number of comparisons (5) and returns a Bonferroni corrected *p-*value. We set the level of significance at 0.05.

## Results

### Metabolic rates

**Fig. 4:**
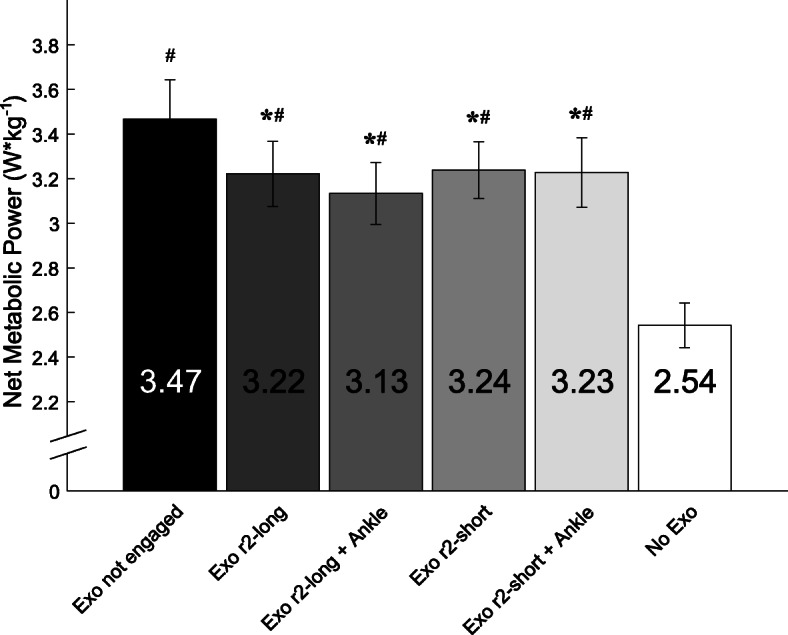
Mean (± SE) Net Metabolic Power required to walk at 1.25 m/s using the exoskeleton (Exo) with no ropes engaged, the exoskeleton with r2-long engaged without and with ankle assistance (Ankle), the exoskeleton with r2-short engaged without and with ankle assistance, and without the exoskeleton. * represents a significant difference compared to Exo not engaged (*p* < 0.05), and # represents a significant difference compared to No Exo (*p <* 0.05)

There was a significant main effect of Condition (F = 29.041, *p* < 0.0001) on net metabolic power. When subjects walked with the spring-ratchet-pawl mechanism engaged, net metabolic power was 6.6–10.7% lower compared to when they walked with the mechanism disengaged (Fig. 4; *p* = 0.025). The greatest reduction in net metabolic power resulted from walking with longer posterior ropes (r2-long) and with r3 engaged to enable ankle assistance, where net metabolic power was 10.7 ± 1.2% lower compared to walking with the mechanism disengaged (*p* = 0.0035). When subjects walked with r3 engaged, and therefore both ankle and knee assistance enabled, their net metabolic power was not significantly different compared to walking with the knee assistance only (*p* = 0.342). Further, we found no significant differences in net metabolic power when subjects walked using longer compared to shorter posterior ropes (*p* = 0.445). When subjects walked with the exoskeleton, their net metabolic power was greater than that measured during normal walking without the exoskeleton. Even for the best exoskeletal condition, net metabolic power was 23% greater compared to normal walking (*p* < 0.0001).

### Kinematic and kinetic parameters

**Fig. 5:**
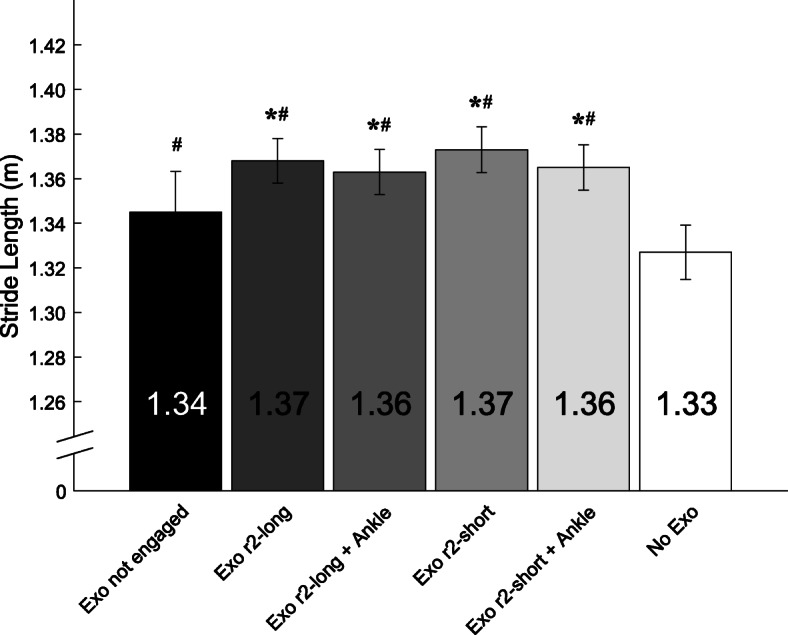
Mean (± SE) Stride Length when subjects walked at 1.25 m/s using the exoskeleton (Exo) with no ropes engaged, the exoskeleton with r2-long engaged without and with ankle assistance (Ankle), the exoskeleton with r2-short engaged without and with ankle assistance, and without the exoskeleton. * represents a significant difference compared to Exo not engaged (*p <* 0.05) and # represents a significant difference compared to No Exo (*p <* 0.05)

There was a significant main effect of Condition for stride length (F = 8.957, *p <* 0.0001). We found that when subjects walked with the exoskeleton, they had 2.7% longer strides compared to walking without the exoskeleton (Fig. 5; *p* = 0.039). We also found that subjects had 1.3–2.0% longer strides when they walked with the exoskeleton disengaged compared to with the exoskeleton engaged (Fig. 5; *p* = 0.045).

**Fig. 6:**
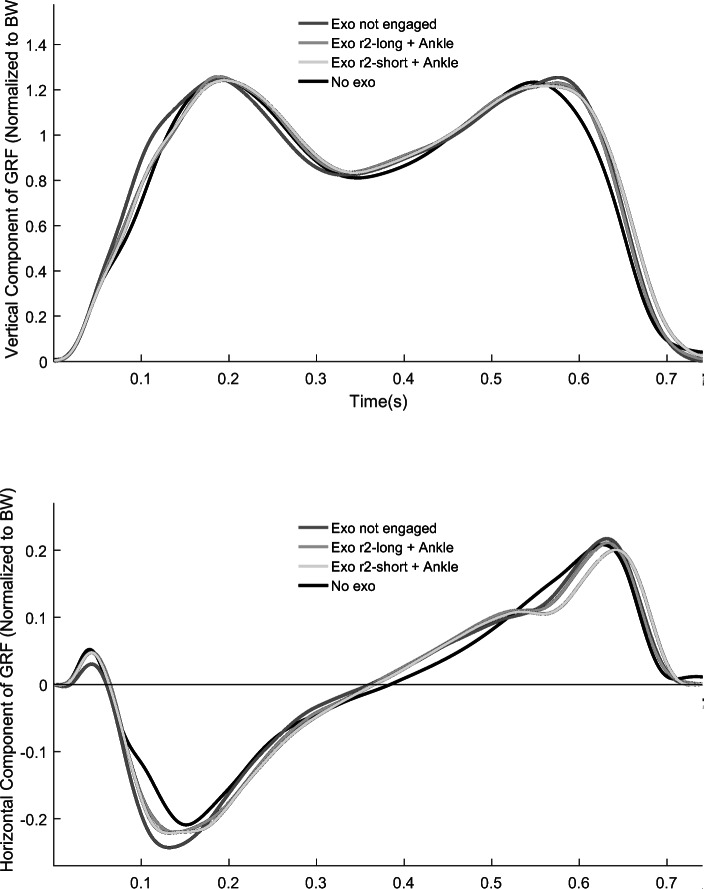
Mean vertical and horizontal ground reaction forces versus time for Subject 5 during the stance phase of walking at 1.25 m/s using the exoskeleton with no ropes engaged (Exo not engaged), the exoskeleton with r-2 long engaged and with ankle assistance (Exo r2-long + Ankle), the exoskeleton with r-2 short engaged and with ankle assistance (Exo r2-short + Ankle), and without the exoskeleton

An overall main effect of Condition was found for contact time (F = 6.763, *p <* 0.0001) We found that ground contact time was ~ 10% longer in all the conditions where subjects walked with the exoskeleton compared to walking without the exoskeleton (Table 1, *p* = 0.036). We found a significant main effect of Condition for the second peak vertical GRFs (F = 6.781, *p <* 0.0001) and peak horizontal propulsive GRFs (F = 5.811, *p* < 0.001). We found that the second peak vertical GRF was 2–3% lower when subjects walked with the exoskeleton disengaged compared to with the exoskeleton engaged (Fig. 6; *p* = 0.018). Additionally, the second peak vertical GRF was 2.63–3.42% lower when subject walked without the exoskeleton compared to with the exoskeleton (*p* = 0.004). There were no significant differences in the second peak vertical GRF between conditions with the exoskeleton engaged with r2-long compared to r2-short (*p* = 0.979) and between the conditions with both knee and ankle assistance enabled compared to knee assistance only (*p* = 0.392). Peak horizontal propulsive GRF was between 5.70 and 9.58% lower in the condition without the exoskeleton compared to walking with the exoskeleton (*p* = 0.015). We found no significant effect of Condition for first peak vertical GRFs (F = 1.038, *p* = 0.413) and peak horizontal braking GRFs (F = 2.058, *p* = 0.099). A significant main effect of Condition was found for step width (F = 4.648, *p* = 0.002). We found that step width was ~ 21% greater in all the conditions where subjects walked with the exoskeleton compared to walking without the exoskeleton (Table 1, *p* = 0.001). There were no significant differences in step width between any of the exoskeleton conditions (*p* > 0.05).

**Table 1:** Mean (± SE) Stride length, contact time, 1st peak vertical ground reaction force (GRF), 2nd peak vertical GRF, peak horizontal propulsive GRF, peak horizontal braking GRF, and step width when subjects walked at 1.25 m/s using the exoskeleton (Exo) with no ropes engaged, the exoskeleton with r2-long engaged without and with ankle assistance (Ankle), the exoskeleton with r2-short engaged without and with ankle assistance, and without the exoskeleton. * represents a significant difference compared to Exo not engaged (*p* < 0.05) and ^#^ represents a significant difference compared to No Exo (*p <* 0.05)

	Exo not engaged	Exo r2-long	Exo r2-long + Ankle	Exo r2-short	Exo r2-short + Ankle	No Exo
**Stride length (m)**	1.34 ± 0.03^#^	1.36 ± 0.03*^#^	1.36 ± 0.03*^#^	1.36 ± 0.03*^#^	1.36 ± 0.03*^#^	1.33 ± 0.04
**Contact time (s)**	0.642 ± 0.02^#^	0.639 ± 0.01^#^	0.642 ± 0.01^#^	0.643 ± 0.01^#^	0.640 ± 0.02^#^	0.582 ± 0.01
**1st peak vertical GRF (N)**	1.13 ± 0.09	1.13 ± 0.09	1.13 ± 0.09	1.13 ± 0.09	1.13 ± 0.08	1.10 ± 0.09
**2nd peak vertical GRF (N)**	1.11 ± 0.09^#^	1.13 ± 0.09*^#^	1.14 ± 0.08*^#^	1.13 ± 0.08*^#^	1.13 ± 0.08*^#^	1.10 ± 0.09
**Peak horizontal propulsive GRF (units of BW)**	0.22 ± 0.01^#^	0.22 ± 0.01^#^	0.21 ± 0.01^#^	0.21 ± 0.01^#^	0.21 ± 0.01^#^	0.20 ± 0.01
**Peak horizontal braking GRF (units of BW)**	−0.22 ± 0.04	−0.21 ± 0.04	−0.22 ± 0.04	−0.21 ± 0.04	−0.21 ± 0.04	−0.20 ± 0.05
**Step width (m)**	0.192 ± 0.01^#^	0.184 ± 0.01^#^	0.183 ± 0.01^#^	0.178 ± 0.01^#^	0.173 ± 0.01^#^	0.152 ± 0.01

## Discussion

The aim of our study was to assess the metabolic cost of using a passive-elastic exoskeleton in parallel with the knee and ankle joints for walking. Use of this exoskeleton, which resists knee extension during the late leg swing phase, did not reduce net metabolic power compared to walking without the exoskeleton; results that do not support our first hypothesis. Also, our second hypothesis was not supported because subjects wearing the exoskeleton with ankle plantarflexion assistance enabled did not have lower net metabolic power compared to walking without the exoskeleton. However, when subjects walked using the exoskeleton with the springs engaged, their net metabolic power was lower compared to when the exoskeleton springs were not engaged. The greatest reduction in net metabolic power was 10.7% and occurred when subjects walked with longer posterior ropes and ankle assistance enabled in the exoskeleton compared to with the exoskeleton not engaged. There were no statistical differences in net metabolic power between exoskeleton conditions with ankle plantarflexion enabled and those without ankle plantarflexion enabled, but with passive knee extension resistance.

An exoskeleton that resists knee extension and stores energy during the late swing phase can reduce net metabolic power during walking compared to an exoskeleton with no assistance provided. Net metabolic power was reduced when the posterior rope (r2) was attached, and therefore only the knee mechanism was engaged. Thus, these results suggest that it is possible to reduce the metabolic cost of walking by only resisting knee extension during the late swing phase, with or without providing ankle plantarflexion assistance. The exoskeleton resists knee extension during the late swing phase and works as a mechanical brake to reduce the speed of the swinging leg prior to heel strike. This deceleration is accompanied by energy dissipation, which corresponds to negative peak knee power performed by the knee flexor muscles (K4; Fig. 1) during level ground walking. The activation of these muscles during the late swing phase causes deceleration of the leg. Our results suggest that the resistance provided by the exoskeleton for knee extension during late swing could reduce the eccentric activation of the knee flexor muscles. Reducing the activity of these muscles could thereby decrease the net metabolic power required for walking. Previous studies using walking exoskeletons have measured the muscle activity of the ankle plantar-flexor muscles with surface electromyography and found a correlation between decreased muscle activation and metabolic power, although some muscle activations are not correlated to metabolic power, likely due to differences in muscle morphology and function [3, 29]. Future research is needed to determine the effects of using the current exoskeleton on the muscle activity of the ankle plantar-flexor and knee flexor muscles (biceps femoris, semitendinosus and semimembranosus) during level ground walking.

Use of recently designed exoskeletons reduce metabolic cost compared to normal walking [3, 25] or compared to walking without the assistance mechanism enabled [26–28, 30, 31] by providing assistance during ankle plantarflexion. Several powered exoskeletons have focused on supplying plantarflexion torque at the ankle by providing positive mechanical power during walking [26–30]. Sawicki & Ferris [27] designed a powered walking exoskeleton that assisted ankle plantarflexion with an actuation system controlled using soleus electromyography. In a similar way, Malcolm et al. [30] used a pneumatic powered exoskeleton that assists ankle plantarflexion during walking and compared different actuation timings predicted using a mathematical model. Both studies found significant reductions in net metabolic power when subjects walked with the powered exoskeleton compared to the unpowered condition. Although these results are promising, these devices were limited because they required power from a battery or needed to be tethered to an external power supply. Other studies show that storing and returning energy during ankle joint negative and positive power phases can further reduce the metabolic cost of level-ground walking compared to walking without an exoskeleton [3, 25] or walking with the assistance mechanism disabled [35]. Lee et al. [35] controlled the level of assistance during ankle joint negative and positive power phases with a powered exosuit and found that subjects had a significantly lower metabolic cost of walking compared to walking with the unpowered exosuit (up to 14.9%). However, this exosuit was tethered to an actuation system and power supply [35] and thus may not be practical in real-world situations. This limitation was overcome by Collins et al. [3] who designed an untethered passive-elastic exoskeleton that stored energy during ankle dorsiflexion and returned it during ankle plantarflexion. Similar to the device of Lee et al., subjects had lower metabolic cost when using the exoskeleton, which assisted ankle joint negative and positive power phases, compared to walking without an exoskeleton [3]. The untethered passive-elastic exoskeleton proposed in the present study was designed to provide assistance during two different phases of the gait cycle. Specifically, it stores energy from knee joint negative power at the end of the swing phase and then provides positive ankle joint plantarflexion power during late stance. To the best of our knowledge, no previous study has considered this approach to build an unpowered exoskeleton aimed at reducing the metabolic cost of walking. Our results indicate that the exoskeleton can reduce metabolic power due to both mechanisms. For example, only assisting the knee joint negative power phase during walking reduced metabolic power.

**Fig. 7:**
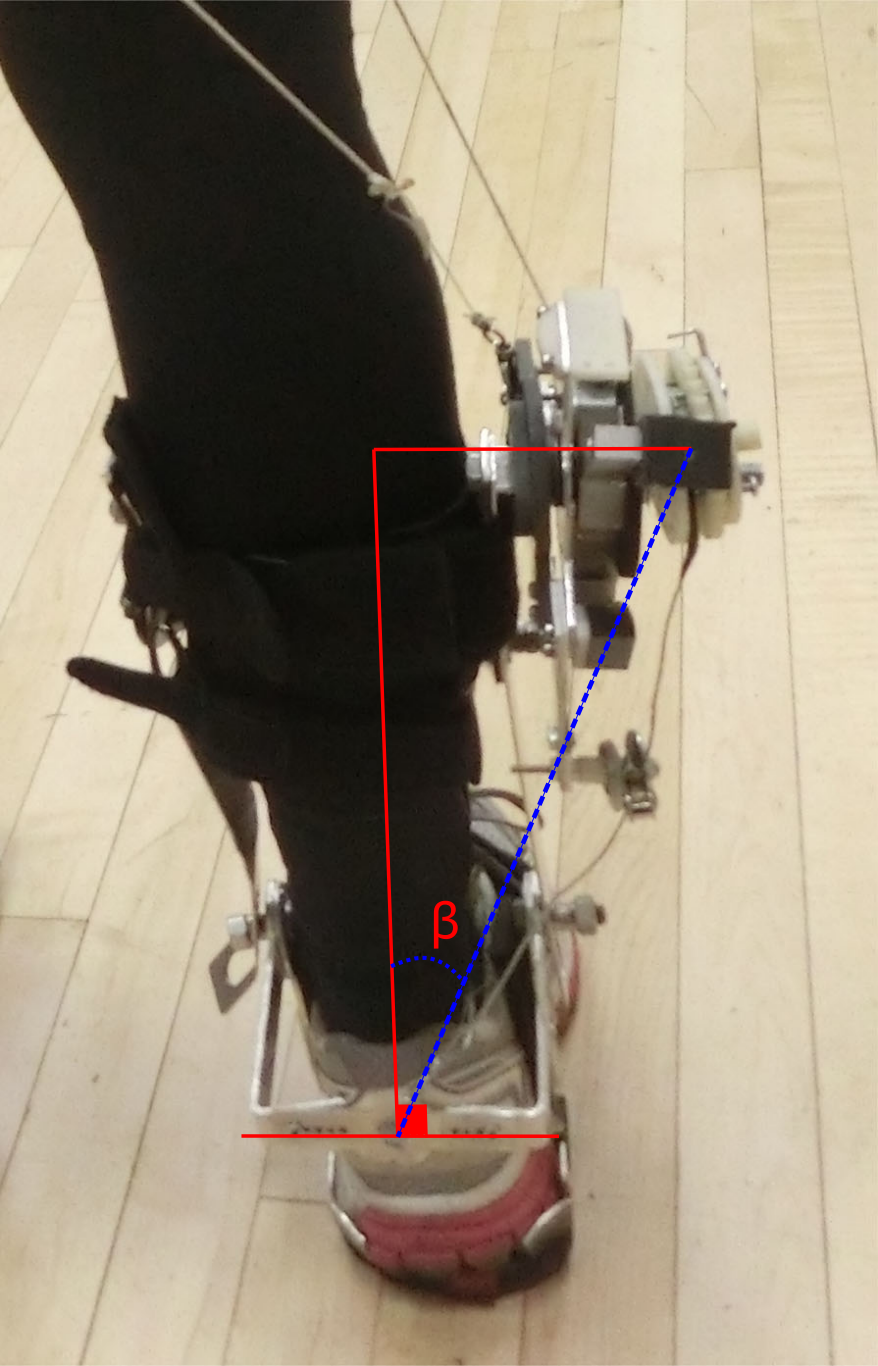
Detailed view of the exoskeleton from the back illustrates that the rope r3 (blue dashed line) was not well-aligned with the ankle frame attachment (red lines are at 90° angles). The engagement of r3 enables ankle plantarflexion assistance, but it is likely that energy is lost due to the angle (β = ~ 45°) of the rope with respect to the shank

We did not measure lower limb segment kinematics or kinetics during the experimental trials, but did record video (Casio, EXILIM EX-ZR1000, Tokyo, Japan, 480 Hz) from all subjects to determine appropriate rope lengths. We used this video to estimate the plantarflexion torque provided by the exoskeleton by determining the approximate angle between the arms of the block (Fig. 2) and a fixed point on the upper frame immediately before toe-off and heel-strike. We used the angle to determine the spring compression at heel strike and compared it to toe-off. The average angle from at least 10 strides for each subject was 0.35 ± 0.05 rad. Since the angular spring stiffness was 17.45 Nm/rad, we estimated that the springs within the exoskeleton provided 6.11 Nm of torque per step. Because rope r3, which assists ankle plantarflexion, is not well-aligned with the ankle frame (Fig. 7), we assume that only a portion of this stored torque is transferred to the ankle frame. Due to the width of the knee apparatus, r3 pulled at about 45° (0.785 rad) with respect to its origin fixed on the posterior case of the knee apparatus. Thus, we estimate that the springs provide approximately 4.3 Nm of torque per step to the ankle frame. The exoskeleton used by Collins et al. (2015) provided approximately 12 Nm of torque for ankle plantarflexion (~ 0.17 N-m/kg body mass) [3]; a higher torque than the estimated torque provided by the current exoskeleton. Thus, it is likely that the current exoskeleton did not provide enough torque at the ankle frame to assist plantarflexion. The inefficient transfer of energy to the ankle frame represents a potential limitation of the current exoskeleton. The use of different materials and improvements in the design of the exoskeleton could reduce the weight of the device and improve the mechanism of energy transfer to the ankle joint. These changes could allow the user of the exoskeleton to further reduce the metabolic power required for walking.

A few potential limitations may have influenced our results. We found no significant differences in metabolic power between walking with ankle plantarflexion enabled and with only the knee extension passive resistance (without the assistance of ankle plantarflexion). This indicates that we may not have optimized the timing and magnitude of ankle plantarflexion power. The ropes r1, r2 and r3 served as mechanical triggers that directly affected the timing of the engagement and disengagement of the springs. It is possible that the adjustments to these rope lengths led to ineffective timing of the storage and release of elastic energy. Future studies are warranted to optimize the timing of elastic energy storage and return. Further, the rope that enabled ankle plantarflexion was not well-aligned with the ankle frame attachment (Fig. 7). Due to the width of the knee apparatus, r3 pulled at ~ 0.785 rad (45°) with respect to its origin fixed on the posterior case of the knee apparatus. Thus, there was likely energy loss within the exoskeleton from the knee to the ankle. Mooney et al. [25] emphasized the importance of developing exoskeletal mechanisms that are closely aligned with the biological joints to prevent undesired forces and translations as well as preserve the amount of torque they can apply [25]. Future designs should address this issue by using pulleys to align the proximal attachment of r3 to the attachment on the ankle frame.

Only the shank braces of the exoskeleton could be adjusted to each participant. Although none of the subjects expressed that they were uncomfortable while walking with the exoskeleton, future device designs that adjust stiffness and length to maximize participants’ comfort and minimize energy loss at the interface between the device and person could further improve metabolic cost during walking. Recent studies have introduced soft exoskeletons as an alternative to rigid exoskeletons to improve the comfort and fit of the device as well as prevent any restriction of the wearer’s movement [31, 32, 35, 41]. These soft exosuits could be particularly suitable to assist walking because they have a lower distal moment of inertia. Furthermore, a soft exoskeleton could be designed to adjust to a wider range of subject sizes and generate torques intrinsically aligned with the biological joints.

Prior to data collection, subjects walked using the exoskeleton for 21 min. Moreover, they performed five 7-min experimental walking trials wearing the exoskeleton. Previous studies show that adaptation time could potentially affect metabolic rates [42, 43]. Thus, it may be possible that with prolonged exposure to using the exoskeleton, subjects may have adopted locomotor strategies that could potentially affect metabolic rates. Minimizing mechanical work, as suggested by Selgrade et al. [42], could represent one of the potential mechanisms involved in the adaptation process. Future studies are needed to determine how and to what extent adaptation time contributes to altering the metabolic and biomechanical demands of walking with an exoskeleton.

The weight of the exoskeleton (1.4 kg per leg) may have resulted in an increased net metabolic power during walking compared to walking without the device. Based on previous studies, adding mass to the lower leg incurs higher metabolic energy expenditure during walking [6, 34]. Browning et al. [34] found that the moment of inertia of the leg and metabolic power increased linearly and proportionally with load when mass was added distally to the lower leg [34]. Based on their findings, we predict that the moment of inertia of the leg would increase by approximately 28% and net metabolic power would increase by approximately 24% with 2.8 kg added distally to the lower legs compared to normal walking. However, when subjects walked with the exoskeleton disengaged, net metabolic power increased by 35% compared to normal walking. This discrepancy could be due to the way the exoskeleton interfaced with the lower leg and how the mass was placed laterally to the lower leg (Fig. 7). The distal placement of the mass of the exoskeleton could have further increased moment of inertia and, thus, net metabolic power. However, the increased distal moment of inertia may have improved the elastic energy storage of the knee exoskeleton system. There is likely a compromise between the estimated 24% increase in metabolic power due to the 1.4 kg added to each lower leg and the potential decrease in metabolic power due to the increased inertia of the lower leg, which could have improved the knee exoskeleton system. However, future designs that replace heavier components with parts made of lighter materials or that move the weight of the device more proximally and/or closer to the lower leg may further reduce the metabolic cost of walking.

The use of a passive-elastic exoskeleton resulted in longer stride lengths during level-ground walking compared to walking without the exoskeleton. We found that when subjects walked using the exoskeleton, stride length was on average 2.7% longer than walking without the exoskeleton. Average preferred stride length was 1.33–1.37 m for all conditions, values consistent with those reported previously where individuals walked at a fixed speed of 1.25 m/s [9]. Umberger et al. [17] found that net metabolic power increased when stride frequency was 10% lower or higher than the average preferred value. Although statistically significant, the stride length differences that we found when subjects used the exoskeleton represent a negligible deviation from optimal stride length and therefore may not be responsible for differences in net metabolic power during walking. It is unclear why stride length changed when subjects walked with the exoskeleton. The changes in stride length could have resulted from the exoskeleton mass added distally to the legs, and therefore increased the moment of inertia. Browning et al. [34] reported that stride length increased by 6 and 11% when 4 and 8 kg were added to each foot, respectively. Assuming a linear relationship between added mass and stride length, we estimate that stride length would increase by 1.75% when 1.4 kg is added to each foot, which is the mass of the exoskeleton. When subjects used the exoskeleton, stride length increased by 2.7% on average. However, the placement of the exoskeleton mass on the lower leg compared to the added mass at the foot may explain the difference in stride length.

Our results indicated that walking with the exoskeleton resulted in a ~ 21% greater step width compared to walking without the exoskeleton, which may have increased metabolic power during walking with the exoskeleton compared to without. Using the model presented in Donelan et al. [19], we predict that metabolic power should increase ~ 6% due to increased step width when walking with the exoskeleton compared to normal walking. However, when subjects walked with the exoskeleton their metabolic power increased by 28% on average compared to without the exoskeleton. As stated previously, this discrepancy could be related to the way the exoskeleton interfaced with participants’ lower limbs and the difference in step width may reflect a compensation mechanism in response to the external load and its placement on the leg.

## Conclusions

We designed a passive-elastic exoskeleton that resists knee extension during the late swing phase and stores this energy within springs, and then returns this elastic energy to assist ankle plantarflexion during the late stance phase. We found that use of this exoskeleton with the springs engaged reduced net metabolic power during walking compared to with the springs disengaged. Future studies are needed to optimize the design and elucidate the underlying biomechanical and physiological effects of using an exoskeleton that acts at the knee and ankle. Moreover, addressing and improving the exoskeletal design by reducing and closely aligning the mass of the exoskeleton could further improve the metabolic cost of walking.

## Data Availability

The datasets used and/or analyzed during the current study are available from the corresponding author on reasonable request.

## Abbreviations

k: Angular stiffness
GRF: Ground reaction force
A1, A2: Positive and negative peaks of ankle joint mechanical power (see Fig. 1)
K1, K2, K3, K4: Positive and negative peaks of knee joint mechanical power (see Fig. 1)
r1: Rope linking the anterior belt to the mechanical apparatus (see Fig. 2)
r2: Rope linking the posterior belt to the mechanical apparatus (see Fig. 2)
r3: Rope linking the mechanical apparatus to the ankle frame (see Fig. 2)
SE: Standard error
r2-short, r2-long: Two different r2 rope configurations, where the short rope length was 1 cm shorter than the long rope length
Exo: Indicates trials when the user was wearing the exoskeleton with no ropes engaged or assistance provided (see Fig. 4)
Ankle: Indicates trials when the user was wearing the exoskeleton with r2-long engaged without and with ankle assistance (see Fig. 4)
